# Multifunctional Role of Lipids in Modulating the Tumorigenic Properties of 4T1 Breast Cancer Cells

**DOI:** 10.3390/ijms23084240

**Published:** 2022-04-11

**Authors:** Yuanyuan He, Somayeh Rezaei, Raimundo Fernandes de Araújo Júnior, Luis J. Cruz, Christina Eich

**Affiliations:** 1 Translational Nanobiomaterials and Imaging (TNI) Group, Department of Radiology, Leiden University Medical Center, 2333 ZA Leiden, The Netherlands; y.he@lumc.nl (Y.H.); s.rezaei@lumc.nl (S.R.); fernandes.araujo@ufrn.br (R.F.d.A.J.); 2 Postgraduate Program in Health Science, Morphology Department, Bioscience Center, Federal University of Rio Grande do Norte (UFRN), Natal 59064-720, Brazil; 3 Cancer and Inflammation Research Laboratory (LAICI), Postgraduate Program in Functional and Structural Biology, Department of Morphology, Federal University of Rio Grande do Norte (UFRN), Natal 59064-720, Brazil

**Keywords:** tumor-associated macrophages, M2 macrophages, palmitic acid, ceramide, sphingomyelin, docosahexaenoic acid, immunomodulation, breast cancer

## Abstract

Tumor growth and progression are linked to an altered lipid metabolism in the tumor microenvironment (TME), including tumor cells and tumor-associated macrophages (TAMs). A growing number of lipid metabolism targeting drugs have shown efficacy in anti-tumor therapy. In addition, exogenously applied lipids and lipid analogues have demonstrated anti-tumor activities in several cancers, including breast cancer. In this study, we investigated the anti-tumor efficacies of the natural lipids palmitic acid (PA), sphingomyelin (SM), ceramide (Cer) and docosahexaenoic acid (DHA) on breast cancer cells. All tested lipids reduced the malignancy of breast cancer cells in vitro by impairing cell proliferation, migration and invasiveness. PA showed superior anti-tumor properties, as it additionally impaired cancer cell viability by inducing apoptosis, without affecting healthy cells. Co-culture experiments further demonstrated that Cer and PA reduced the immunosuppressive phenotype of M2 macrophages and the M2 macrophage-promoted the epithelial–mesenchymal transition (EMT) and migration of breast cancer cells. At the molecular level, this coincided with the up-regulation of E-cadherin. Our results highlight a powerful role for exogenously applied PA and Cer in reducing breast cancer tumorigenicity by simultaneously targeting cancer cells and M2 macrophages. Our findings support the notion that lipids represent alternative biocompatible therapeutic agents for breast cancer.

## 1. Introduction

Breast cancer is the most commonly diagnosed cancer among women, with an estimated 2.3 million new cases and 685,000 deaths worldwide [1]. Although some great progress has been obtained in breast cancer treatment, there are still many patients suffering from relapse and metastasis, leading to high mortality [2,3]. As a heterogeneous and aggressive disease [4], breast cancer cells have no inhibitory effect on their division and proliferation [5]. In addition, the reduced adhesion between cancer cells leads to invasion into healthy tissues and metastasis [6]. Initially, scientists’ efforts to find novel treatments for breast cancer focused on attacking tumor cells [5,7,8] however, in recent years more and more studies support the notion that the tumor microenvironment (TME) is involved in cancer progression and metastasis [9].

As a component of the TME, tumor-associated macrophages (TAMs) play an important role in regulating the interaction between cancer cells and the immune system [5,10]. While M1 macrophages possess pro-inflammatory, anti-microbial and anti-tumoral activities [11,12,13], M2 macrophages are involved in tissue remodeling, angiogenesis and wound healing [14]. However, in the TME, TAMs resemble M2-polarized macrophages and promote the growth and metastasis of cancer cells, and are thought to be responsible for the development of resistance to multiple treatments [8,15]. Importantly, studies have shown that M2 macrophages can be “re-educated” towards M1 macrophages to kill tumor cells [16,17]. Therefore, exploring how to repolarize M2 macrophages into M1 macrophages in the TME is a hot spot of current breast cancer immunotherapy research [18].

Lipids belong to a class of amphiphilic or hydrophobic small molecules [19] that are important components of cell membranes and signaling pathways. As a substrate for energy metabolism, lipids play a critical role in regulating cell development, differentiation, apoptosis and other essential cellular activities [20,21]. More recently, it has been shown that an abnormal lipid metabolism is closely associated with the development and progression of cancer [22]. Highly proliferating cancer cells require large amounts of lipids as biofilm support [23]. In addition, increased endogenous fatty acid biosynthesis, fatty acid oxidation and cholesterol accumulation are linked to a variety of oncogenic processes, including cell proliferation, migration and chemoresistance formation [19].

In breast cancer, lipid metabolism is regulated by both endogenous lipogenesis and extracellular lipids [24]. For example, Marino et al. demonstrated that genes involved in lipid metabolism and adipogenesis are simultaneously upregulated in breast tumors [25]. Higher levels of fatty acid synthase expression have been reported in breast cancer cells that overexpress HER2 [26]. Drug resistance and lipid metabolism are also interlinked, as ectopic expression of fatty acid synthase can lead to drug resistance [27]. In addition, the mammary glands contain a large number of adipocytes that can transfer stored lipids to breast cancer cells [19,28].

Several lipids were reported to modulate the viability and function of cancer cells. Ceramides, fatty acid derivatives of sphingosine bases, are lipids with pro-apoptotic and anti-proliferative effects [29]. In cancer combinational therapies, promotion of ceramide (Cer) synthase activity has been shown to increase intracellular Cer levels and to promote apoptosis in tumor cells [30,31]. At the molecular level, Cer causes cell death through several mechanisms, for example by enhancing the activity of pro-apoptotic membrane molecules (e.g., CD95) [32]. Sphingomyelin (SM), an essential component of the plasma membrane, is involved in the regulation of cell membrane stability and secretory activities [33]. Results from various cancer studies demonstrate that SM promotes cancer development, immune evasion and metastasis by regulating cancer cell proliferation and invasion [34,35,36]. Sphingolipid levels have been reported to be higher in breast cancer compared to normal breast tissue [37]. In contrast, a more recent study identified SMs as key secreted factors that possess natural cancer-suppressing mechanisms [38]. In vivo, Cer is converted to SM by the addition of a phosphorylcholine headgroup by SM synthase [39], while sphingomyelinase can reduce the levels of SM by conversion of SM to ceramide (Cer) [40,41]. Tumor progression has been linked to a disturbed metabolic balance of SM and Cer [42]. Palmitic acid (PA), a saturated long-chain fatty acid, is an important metabolite of fatty acid synthase and has been reported to inhibit the invasiveness of different types of cancer cells [43,44]. In highly migratory ER-negative breast cancer cells, PA is involved in lipid signaling and plays an important role in the induction of apoptosis [45,46]. As a polyunsaturated fatty acid [47], docosahexaenoic acid (DHA) has been shown to affect cell proliferation by prolonging the cell cycle during the G2/M transition [48], and by inducing apoptosis through various pathways [49], including an increase in Bcl-2, caspase-3 and procaspase-8 activity [50,51]. Previous in vitro and in vivo studies demonstrated that DHA plays a major role in the anti-tumor properties of mammary glands [49,52,53].

Lipids have also been reported to regulate TAM generation, differentiation and function in the TME. In particular, lipid accumulation in TAMs is critical for TAM polarization and tumor proliferation [54]. For example, Wu et al. demonstrated that oleic acid promotes the polarization of bone marrow-derived macrophages to an M2-like phenotype, which prevented tumor cells from evading immune surveillance [55]. In the TME, Cer plays a key role in mediating apoptosis of bone marrow-derived macrophages (BMDMs) [56]. Moreover, Li et al. showed that injection of nano-lipid-coated C-6 ceramide in a tumorigenic mouse model not only reduced the number of TAMs, but also promoted their antitumor immune activity [57]. SM is one of the important lipids that regulate inflammation [58] and the inflammatory response of macrophages [59,60]. PA upregulates the expression of pro-inflammatory molecules in macrophages by amplifying LPS signaling [61], and we recently demonstrated that PA decreased the M2 phenotype in macrophages [44]. DHA is one of the precursors that regulates inflammatory regressive molecules and can affect the M1/M2 balance [62]. These findings indicate that the lipids SM, Cer, DHA and PA possess immunomodulatory properties.

As the literature provides evidence that the lipids Cer, DHA and PA display anti-tumorigenic properties in different types of cancer, we explored whether these lipids could be used to treat breast cancer through multiple modes of action: First, by inhibition of tumor cell growth and secondly, by reversing the immunosuppressive milieu in the TME by promoting the M1 phenotype with antitumor activity. To this end, we thoroughly investigated the antitumor properties of PA, Cer, DHA and SM in vitro in breast cancer cells, and upon co-culture with soluble factors secreted by M2 macrophages treated with lipids. Our results show that PA specifically promoted the apoptosis of cancer cells, while non-toxic doses of PA, Cer, SM and DHA induced the repolarization of M2 macrophages towards the M1 type. In addition, we found that PA, Cer, DHA and SM reduced the proliferative, migratory and invasive properties of breast cancer cells.

## 2. Results

### 2.1. Effect of Cer, DHA, SM and PA on the Cellular Activity of 4T1 Breast Cancer Cells and Macrophages

**Figure 1:**
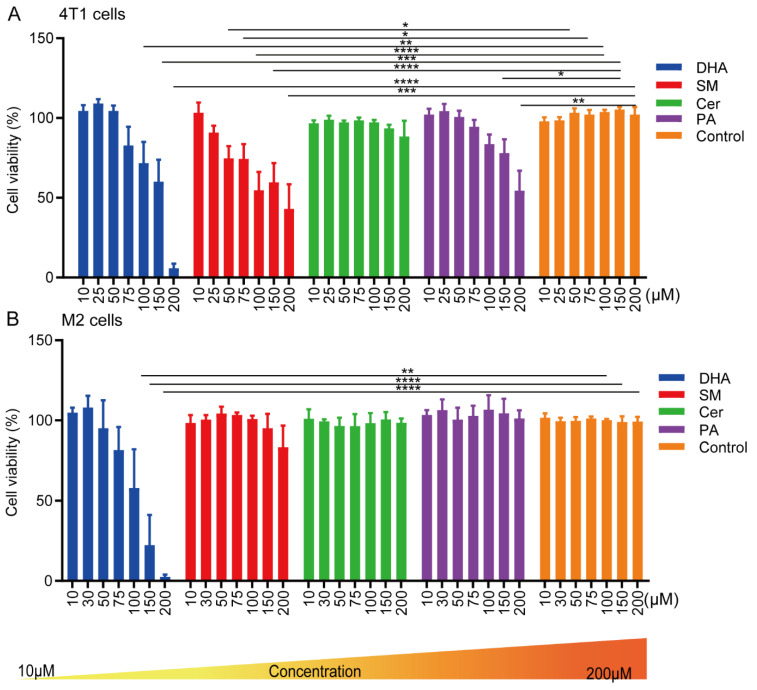
Cell viability of (**A**) 4T1 and (**B**) M2 cells after treatment with SM, DHA, Cer or PA for 48 h. 4T1 and M2-macrophages were treated with increasing concentrations (10–200 μM) of SM, DHA, Cer, or PA. After 48 h, the cell viability was assessed by MTS assay. The data represent the mean ± SEM of 6 independent experiments. All *p*-values were compared to the respective control cells using a two-way ANOVA test. * *p* ≤ 0.0272; ** *p* ≤ 0.007; *** *p* ≤ 0.0003; **** *p* ≤ 0.0001.

First, we assessed the effect of different concentrations of PA, SM, Cer and DHA on the metabolic activity and viability of 4T1 breast cancer cells and M2 type-differentiated RAW 264.7 macrophages, representative of the dominant cell types in the TME, by an MTS assay (Figure 1A,B). In addition, to investigate whether the lipids differentially affected macrophages dependent on their polarization state, we also performed the experiment on non-differentiated (M0) and M1 type RAW 264.7 macrophages (Supplementary Figure S1A,B). DHA had a prominent effect on the metabolic activity of the tested cell lines. In the presence of 75 μM DHA and 48 h after culture, the metabolic activity of 4T1, M0, M1 and M2 cells decreased by 21.3%, 55.9%, 49.6% and 15.3%, respectively, compared to the control cells (Figure 1A,B and Supplementary Figure S1A,B). Higher doses (100–200 μM) further decreased the metabolic activity of all cell lines. In contrast to DHA, treatment of 4T1 cells with 25–200 μM of SM for 48 h decreased the cellular activity from 94.1% to 43.0%, but had almost no effect on the viability of macrophages (Figure 1A,B and Supplementary Figure S1A,B). Similarly, 150 μM of PA significantly affected the metabolic activity of 4T1 cells but had no effect on macrophages. We did not observe any cytotoxic effect of Cer on 4T1 cells and macrophages within the tested concentration range of 10–200 μM. In conclusion, the lipids DHA, SM and PA showed concentration-dependent effects on the metabolic activity of 4T1 cells. Concentrations of DHA of above 75 μM significantly affected the metabolic activity of breast cancer cells and macrophages, while SM (50 μM) and PA (150 μM) induced a reduction in metabolic activity specifically in breast cancer cells, without affecting macrophages.

### 2.2. PA and SM Induce Apoptosis in Breast Cancer Cells

**Figure 2:**
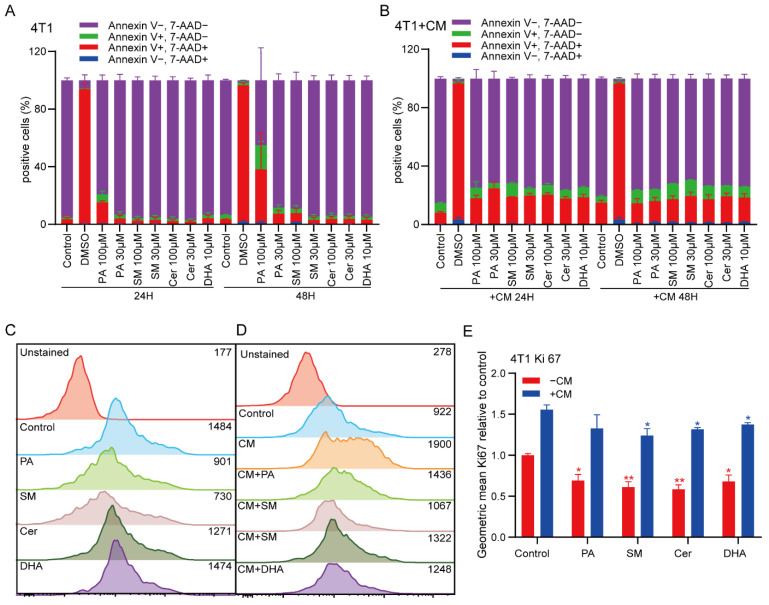
Effect of lipids and CM of M2 macrophages treated with lipids (CM+) on apoptosis and proliferation of 4T1 cells. Annexin-V/7AAD labeling to determine the rate of apoptosis and necrosis in 4T1 cells treated with (**A**) lipids and (**B**) CM+ for 24 h and 48 h. Representative flow cytometry graphs of 4T1 cells 48 h after treatment with (**C**) 30 μM PA, SM and Cer, or 10μM of DHA directly, or (**D**) CM+ 30 μM PA, SM and Cer, or 10μM of DHA. 4T1 cells were stained with the proliferation marker Ki-67 and analyzed by flow cytometry. (**E**) Summary quantification of the mean fluorescent intensity of Ki-67 expression in control, lipid- and CM-treated 4T1 cells. The data represent the mean ± SEM from an average of 3 independent experiments. Statistical significance was determined using Student’s *t*-tests (*n* = 3, * *p* ≤ 0.05; ** *p* ≤ 0.01).

The MTS assay suggested that PA, SM and DHA reduced the metabolic activity of breast cancer cells, but did not report on whether this reduction was caused by cell death or altered proliferative behavior. Thus, we explored whether the lipids induced cancer cell apoptosis and defined the concentration of each individual lipid that did not induce apoptosis for further functional experiments. To this end, we cultured 4T1 cells in the presence of 30 μM and 100 μM PA, SM and Cer. As the MTS data suggested a stronger effect of DHA, we incubated the 4T1 cells with 10 μM DHA. After 24 h and 48 h, 4T1 cells were labeled with Annexin V in combination with the viability dye (7AAD) to discriminate viable cells from apoptotic and necrotic cells by flow cytometry. In line with our MTS results, the data showed that after 24 h of treatment with 100 μM PA, the percentage of Annexin-V positive cells increased from 4.5% in the control group to 20.3%, and to 53.7% after 48 h (control group 6.2%), while 30 μM PA only had a minor effect on the viability of 4T1 cells after 24 h (6.5%) and 48 h (11.1%) (Figure 2A). Similarly, 100 μM SM induced apoptosis in 9.6% of 4T1 cells after 48 h (Figure 2A). M2-polarized TAMs are known to secrete soluble factors, such as cytokines, which contribute to the establishment of an immunosuppressive TME and to promote the metastatic properties of cancer cells. To analyze the effect of soluble factors derived from M2 macrophages on breast cancer cell viability, 4T1 cells were co-cultured with CM of M2-differentiated macrophages for 24 h and 48 h. Addition of CM induced apoptosis in 19.2% of the cells. However, the addition of CM in macrophages treated with lipids (CM+) did not further increase the level of apoptosis in 4T1 cells. In conclusion, our data showed that high doses (100 μM) of exogenous PA, directly added to 4T1 cells, induced apoptosis.

Furthermore, we explored whether the lipids PA, DHA, Cer and SM, and CM+ could affect cancer cell proliferation. To this end, we treated 4T1 cells with low doses of PA, DHA, Cer, or SM (30 μM), or with CM+ PA, DHA, Cer or SM (30 μM) for 48 h, and analyzed the expression of Ki-67 (a proliferation marker) by flow cytometry. Expression of Ki-67 was significantly reduced in 4T1 cells after PA, DHA, Cer and SM treatment compared to the untreated group (Figure 2C–E, * *p* ≤ 0.05, ** *p* ≤ 0.01). When 4T1 cells were co-cultured with CM for 48 h, the expression of Ki-67 was significantly upregulated compared to the untreated control group (Figure 2E). However, when 4T1 cells were treated with CM+ DHA, Cer and SM, Ki-67 expression was significantly decreased, compared to the control group (CM). In addition, we observed a non-significant trend towards reduced proliferation in the CM+PA group (Figure 2C–E). In conclusion, our data show that low concentrations of all lipids did not affect cell viability, but greatly reduced the proliferation of 4T1 cancer cells. Thus, the observed reduction in cell proliferation after treatment with PA (low concentration), SM, Cer and DHA, or CM+ could not be attributed to a reduction in cell viability. All further studies explored the impact of low lipid concentrations that did not induce cell death on cancer cell functions.

### 2.3. Effect of Lipids on the Polarization State of M1 and M2 Macrophages

The ideal anti-cancer drug has anti-tumor properties while simultaneously reducing the immunosuppressive state in the TME. Here, we investigated whether the lipids could also reverse the polarization state of M2 macrophages. To ensure that the reversal of the polarization of M2 macrophages was caused by the action of lipids and not by cytotoxic effects, we performed all experiments in the presence of 30 μM PA, SM and Cer, or 10 μM DHA.

**Figure 3:**
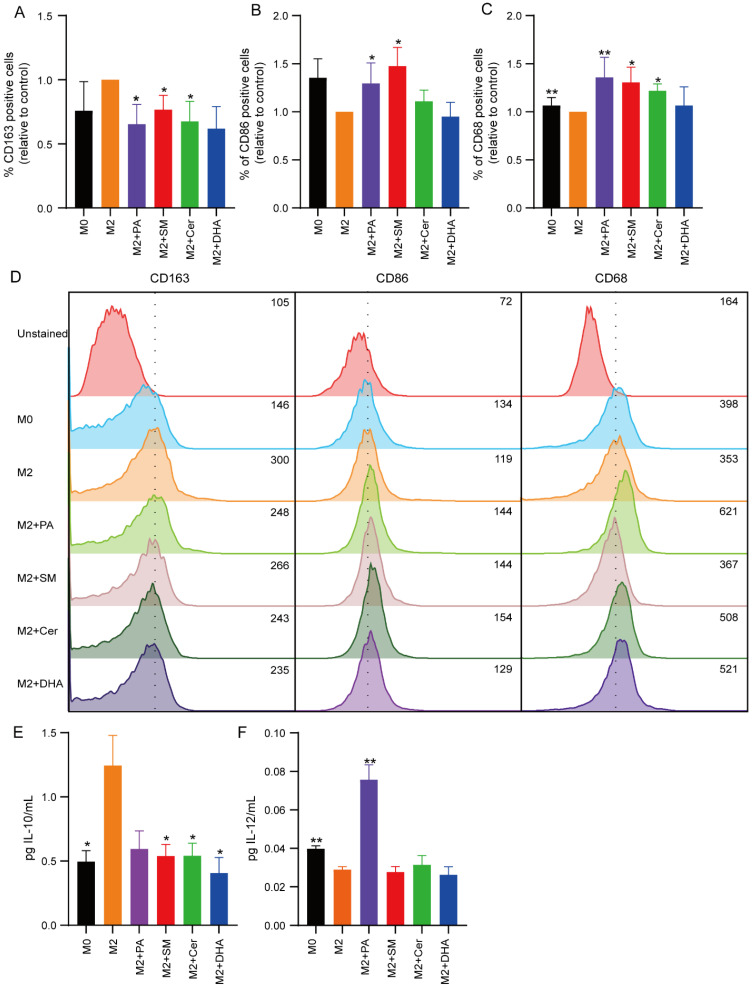
Cer, SM and PA decreased the M2 phenotype in macrophages. Macrophages were polarized towards the M2 type in the presence of 30 μM PA, SM, Cer or 10 μM DHA for 48 h and the percentage of (**A**) CD163, (**B**) CD86 and (**C**) CD68 positive cells were determined by flow cytometry. (**D**) Representative flow cytometry profiles of CD163, CD68, and CD86 expression in M0 cells, M2 control cells, and M2 macrophages after treatment with 30 μM PA, SM, Cer, or 10 μM DHA. The M2 and M1 cytokines IL-10 (**E**) and IL-12 (**F**) from supernatants of M2 control cells and cells treated with 30 μM PA, SM, Cer or 10 μM DHA were measured by ELISA. The data represent the mean ± SEM of 3–5 independent experiments. All p values were compared to control cells by analysis of variance and the Mann-Whitney test, * *p* ≤ 0.05; ** *p* ≤ 0.01.

RAW 264.7 macrophages were polarized to M2 macrophages in the absence or presence of 30 μM PA, SM and Cer, or 10 μM DHA. Subsequently, M1 or M2 macrophage-specific cell surface markers (M1 = CD86, CD68; M2 = CD163) were analyzed on the treated cells by flow cytometry. The results showed that the expression of the M2 macrophage marker CD163 was significantly increased in the IL-4-treated group (control group) (Figure 3A–D), indicating that IL-4 successfully induced the conversion of M0 macrophages to M2-polarized TAMs, whereas the expression of the M1 markers CD68 and CD86 was decreased in M2-polarized macrophages (Figure 3A–D). Compared to the control group, the addition of 30 μM PA during M2 polarization significantly decreased the expression of CD163 (* *p* ≤ 0.05) (Figure 3A), and significantly elevated the expression of CD86 and CD68 (* *p* ≤ 0.05). Similarly, addition of 30 μM Cer significantly decreased the expression of CD163 (* *p* ≤ 0.05) (Figure 3A) and elevated expression of CD68 (* *p* ≤ 0.05), but not CD86 (Figure 3A–D). Surprisingly, addition of 30 μM SM also led to significantly (* *p* ≤ 0.05) elevated expression levels of CD86 and CD68 (Figure 3B,C), and significantly decreased the expression of CD163 (* *p* ≤ 0.05) (Figure 3A).

To further confirm the effect of these lipids on the macrophage polarization state, we collected cell culture supernatants 48 h after treatment and measured the levels of IL-10 and IL-12 cytokines by ELISA to determine the presence of immunosuppressive and pro-inflammatory cytokines, respectively (Figure 3E,F). We observed a trend towards increased expression levels of IL-10 levels in M2 polarized macrophages compared to M0 cells (Figure 3E), while IL-12 levels were significantly decreased (** *p* ≤ 0.01) (Figure 3F). Interestingly, all lipids induced a strong trend towards reduced IL-10 secretion, although the results were not significant. However, in agreement with previous results, treatment with 30 μM PA significantly increased IL-12 levels (** *p* ≤ 0.01) (Figure 3F). As RAW 264.7 cells are immortalized cells that might differ from primary macrophages, we speculated whether BMDMs would show a similar response towards lipids. To this end, we derived macrophages from bone marrow and differentiated the cells towards the M2 type in the presence of IL-4 and IL-13. MTS measurements showed that high doses (≤75 μM) of Cer and DHA reduced the metabolic activity of BMDMs (Supplementary Figure S2A). At non-cytotoxic concentrations, the lipids induced changes in the morphological appearance of the cells (Supplementary Figure S2B). In agreement with the literature, murine BMDMs assumed an elongated phenotype upon stimulation with M2 cytokines [63]. Visual inspection showed that addition of Cer, PA and DHA reduced the degree of elongation, compared to untreated M2 macrophages, whereas SM had no visual effect on BMDM morphology. Quantification of IL-10 and IL-12 cytokines from the cell culture supernatants 48 h after treatment with lipids showed a significant reduction in IL-10 secretion upon treatment with PA, SM, Cer and DHA, and increase in IL-12 secretion upon treatment with PA, compared to untreated M2 BMDMs (Supplementary Figure S2C,D). Thus, these data confirm our findings on RAW 264.7 cells.

In summary, these results confirm and extend our previous findings [44], indicating that PA, Cer and SM reduced the immunosuppressive phenotype of M2 polarized RAW 264.7 cells, but only PA increased the expression of IL-12.

### 2.4. PA, DHA, SM and Cer Reduce the Migratory Properties of 4T1 Cells

**Figure 4:**
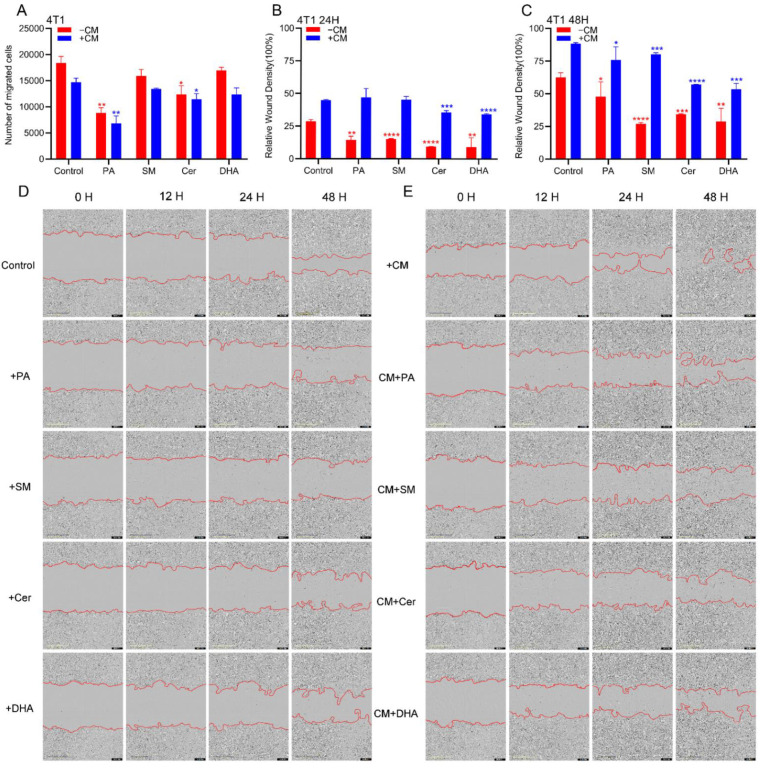
PA, SM, DHA, and Cer inhibited the migration and invasiveness in 4T1 cells. (**A**) The number of viable migrated 4T1 cells after culture with PA, SM, Cer, or DHA, or co-culture with CM of M2 macrophages treated with PA, SM, Cer, or DHA were determined by trypan blue staining. Invasive properties of 4T1 cells (**B**) 24 h and (**C**) 48 h after culture with lipids or co-culture with CM of M2 macrophages treated with lipids were assessed in a wound-healing assay. Representative images of the 4T1 wound area 48 h after treatment with (**D**) PA, SM, Cer, or DHA, or (**E**) with CM+. Images were analyzed by the IncuCyte^®^ system software. All *p* values were analyzed by Student’s t-tests (*n* = 3, * *p* ≤ 0.05; ** *p* ≤ 0.01, *** *p* ≤ 0.001 **** *p* ≤ 0.0001 vs. control).

Metastasis is a major cause of death from breast cancer, so reducing the migration and invasive potential of breast cancer cells is an important objective of breast cancer treatments. Our previous study demonstrated that M2 macrophages can promote the development of EMT in colorectal cancer cells [64]. In this study, we investigated whether PA, DHA, SM and Cer, and CM of macrophages treated with lipids (CM+) could also affect the migratory properties of 4T1 breast cancer cells. For this purpose, 4T1 cells were treated with PA, Cer, SM, or DHA and their migratory properties were assessed in a transwell migration assay. After 48 h, the number of viable 4T1 cells migrating through the pores of the transwell system was quantified by trypan blue labelling. Compared to the control group, 30 μM PA and Cer significantly (*p* ≤ 0.05) inhibited the migration of 4T1 cells (Figure 4A). Next, to analyze the effect of soluble factors derived from M2 macrophages on breast cancer cell migration, we repeated the transwell migration assay by co-culturing 4T1 cells with lipid-treated M2 CM (CM+) (Figure 4A). In the samples treated with 30 μM CM+ PA and 30 μM CM+ Cer, the migration of 4T1 cells was significantly inhibited (*p* ≤ 0.05), compared to the untreated cells.

To determine whether the invasive properties of breast cancer cells were also affected by lipids and/or by CM+, we performed a wound-healing assay using the IncuCyte^®^ system to monitor the wound healing capacity of 4T1 cells in real time (Figure 4B–E). Since in this assay cell proliferation resembles cell migration, 4T1 cells were treated during this experiment with mitomycin C, a cell proliferation inhibitor. Our data showed that after treatment of 4T1 cells with PA, SM, Cer and DHA, there were significantly fewer cells migrating to the wound area after 24 and 48 h, compared to the untreated cells. In contrast to 4T1 cells treated with lipids alone, significantly more cells migrated to the wound area after exposure to M2 CM (Figure 4B,C). However, significantly fewer cells were found in the 4T1 cell wound area after treatment with CM+ lipids, compared to the CM control group (Figure 4D,E). In conclusion, SM, Cer, DHA and PA reduced the invasive properties of 4T1 breast cancer cells, also after CM-promoted increase in wound healing capacity.

### 2.5. Lipids Decreased the Mesenchymal Phenotype in Breast Cancer Cells

**Figure 5:**
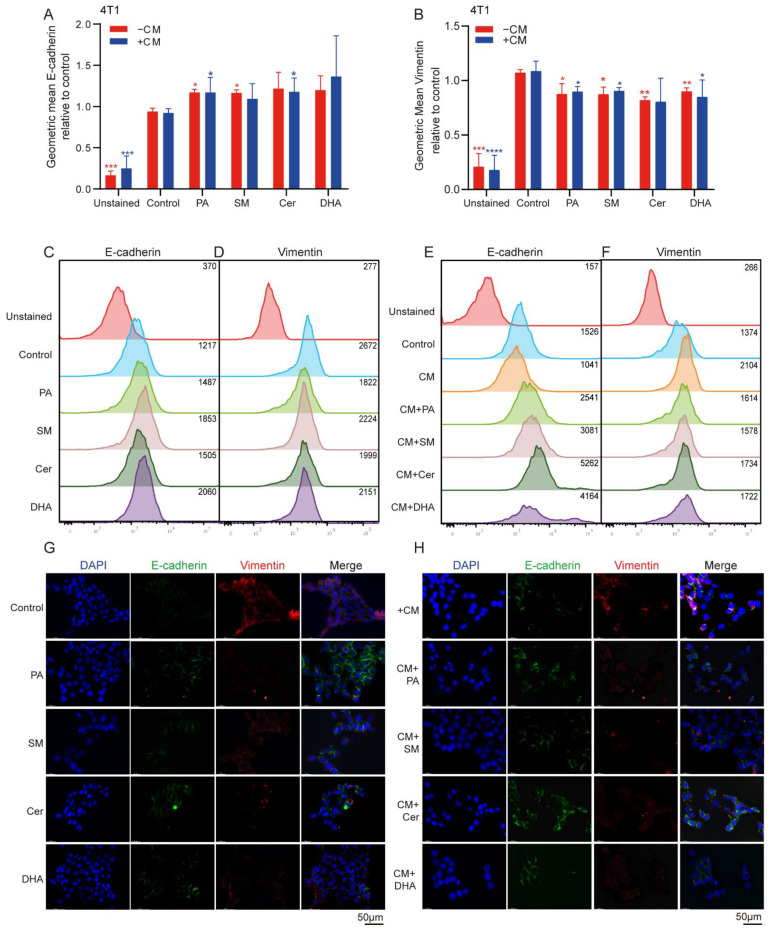
Co-culture of 4T1 cells with CM of M2-polarized TAMs increased the mesenchymal phenotype in breast cancer cells. 4T1 cells were cultured with 30 μM PA, SM, Cer or 10 μM DHA, or co-cultured with CM+ 30 μM PA, SM, Cer or 10 μM DHA for 48 h and analyzed by flow cytometry for (**A**) E-Cadherin and (**B**) vimentin expression. Representative flow cytometry plots of (**C**) E-cadherin and (**D**) vimentin expression on lipid-treated 4T1 cell (**E**) and E-cadherin and (**F**) vimentin expression after treatment with CM+. Immunofluorescence images of 4T1 cells treated with (**G**) 30 μM PA, SM, Cer or 10 μM DHA, or (**H**) co-cultured with CM+ for 48 h, stained for E-Cadherin (green) and vimentin (red) expression. The data represent the mean ± SEM of 3–5 independent experiments. All p values were compared to control cells by analysis of variance and the Mann–Whitney test, * *p* ≤ 0.05; ** *p* ≤ 0.01, *** *p* ≤ 0.001, **** *p* ≤ 0.0001, versus control. Scale bar = 50 μm.

When cancer cells get more invasive, they become more mobile, which is accompanied by a change from an epithelial to a mesenchymal phenotype. Cells with an epithelial phenotype express higher level of E-cadherin, while cells with mesenchymal phenotype are characterized by the down-regulation of E-cadherin and upregulation of vimentin, a cytoskeletal component. To investigate whether lipids could affect the mesenchymal phenotype of 4T1 cells, the cells were cultured alone or in the presence of M2-derived CM, and the expression levels of the epithelial marker E-cadherin and the mesenchymal marker vimentin were analyzed by flow cytometry and an immunofluorescence microscope. Flow cytometric analysis showed that the expression levels of E-cadherin significantly increased (* *p* ≤ 0.05) when 4T1 cells were cultured in 30 μM PA and SM (Figure 5A), while the expression of vimentin slightly decreased after treatment with 30 μM PA, SM, Cer and 10 μM DHA, albeit at significant levels (Figure 5B). Treatment of 4T1 cells with CM of M2 macrophages did not show significant changes in E-cadherin and vimentin expression compared to controls group. However, when 4T1 cells were treated with CM+ 30 μM PA and Cer, we observed a significant increase (* *p* ≤ 0.05) in E-cadherin expression (Figure 5A,E, * *p* ≤ 0.05; ** *p* ≤ 0.01), whereas the expression of vimentin slightly decreased after co-culture with CM+ 30 μM PA, SM and DHA (Figure 5B,F, * *p* ≤ 0.05; ** *p* ≤ 0.01). Thus, PA, Cer, SM and DHA promoted the epithelial phenotype in 4T1 cells by increasing E-cadherin and/or downregulating vimentin expression. Addition of CM increased the mesenchymal phenotype of 4T1 cells; however, treatment with CM+ partially overruled the cell invasion-promoting effects of CM on 4T1 cells. However, only PA increased E-cadherin expression and concomitantly downregulated vimentin expression when added to 4T1 cells directly and as CM+PA.

### 2.6. PA, Cer, SM and DHA Decreased the Mesenchymal Phenotype of 4T1 Cells at the Molecular Level

**Figure 6:**
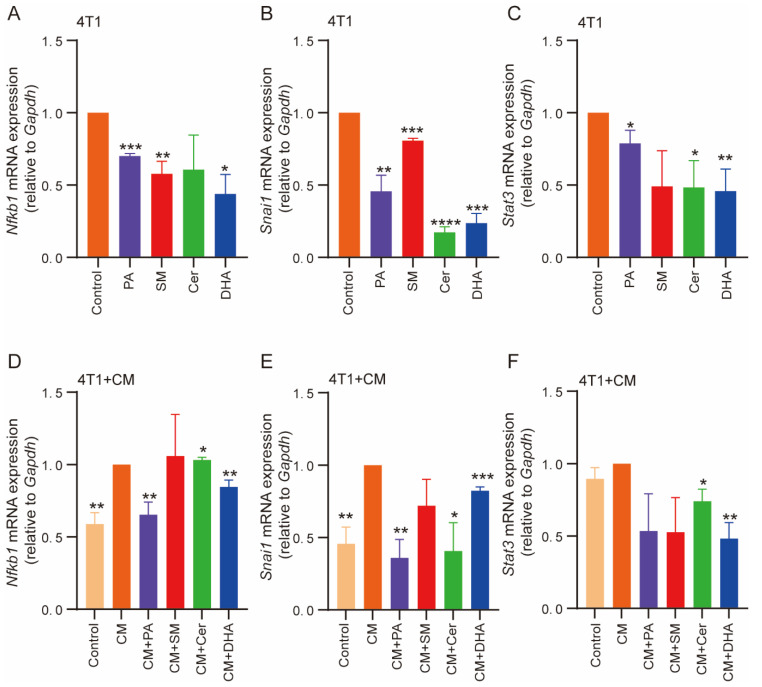
Co-culture of 4T1 cells with lipids or CM+ PA, SM, Cer and DHA decreased the gene expression of inflammation- and apoptosis-related genes in breast cancer cells. mRNA expression of (**A**) *Nfkb1*, (**B**) *Snai1* and (**C**) *Stat3* in 4T1 cells treated with 30 μM PA, SM, Cer or 10 μM DHA. (**D**) *Nfkb1*, (**E**) *Snai1* and (**F**) *Stat3* mRNA expression in 4T1 cells treated with CM+ 30 μM PA, SM, Cer or 10 μM DHA. The data is shown relative to *Gapdh* expression. The data represent the mean ± SEM of 3–4 independent experiments. All *p* values were compared to control by unpaired *t*-test, * *p* ≤ 0.05, ** *p* ≤ 0.01, *** *p* ≤ 0.001, **** *p* ≤ 0.0001.

The high expression of STAT-3 and Snail genes has been reported to contribute to cell proliferation and migration and promote EMT [65]. NF-κB signaling can coordinate many signals that drive proliferation in immune, inflammatory and oncogenic processes [66]. To further compare the role of lipids and the CM of M2-macrophages cultured with lipids in EMT, we analyzed the mRNA expression levels of *Snai1*, *Stat3* and *Nfkb1* by RT-qPCR. The data showed that the mRNA expression levels of *NF-κB1* (Figure 6A) and *Snai1* (Figure 6B) were significantly reduced in 4T1 cells after treatment with PA, SM and DHA, compared with the control. In addition, *Snai1* was also reduced in response to Cer treatment. The mRNA expression level of *Stat3* was significantly decreased after treatment with PA, Cer and DHA (Figure 6C). Next, we verified whether NF-κB protein levels were also reduced by Western blot labeling (Supplementary Figure S3A). The results showed that the expression levels of NF-κB protein were significantly reduced after treatment with PA, SM, Cer and DHA. When 4T1 cells were treated with CM of non-treated M2 macrophages, the mRNA expression levels of *Nfkb1*, *Snai1* and *Stat3* significantly increased, compared to M0 cells (Figure 6D–F). However, upon incubation with CM+ PA, DHA and Cer, the mRNA expression levels of *Nfkb1 and Snai1* significantly decreased (Figure 6D,E). The expression of *Stat3* was significantly reduced in response to treatment with CM+ DHA and Cer (Figure 6F). When 4T1 cells were treated with the CM of untreated M2 macrophages, the expression of NF-κB protein was higher than in cells cultured with the CM of M2 macrophages treated with PA (30 μM), SM (30 μM), Cer (100 μM) and DHA (10 μM) (Supplementary Figure S3B). In conclusion, PA, SM, DHA, and Cer induced molecular changes in signaling pathways associated with oncogenic processes, including EMT. This is consistent with the observed effects of lipids on breast cancer proliferation, migration and invasion, which are likely caused by lipid-induced alterations in underlying signaling pathways.

In summary, all of the evaluated lipids showed anti-tumor properties by impacting on tumor cell viability and functions and inducing the repolarization of M2-type macrophages towards the inflammatory type 1 (Table 1). Overall, PA and SM displayed the greatest anti-tumor properties in 4T1 cells, including the reduction of 4T1 cell viability, as well as immunomodulatory effects by reducing the M2-phenotype in macrophages.

## 3. Discussion

In cancer cells, abnormalities in lipid metabolism, biosynthesis and signaling have been shown to promote the proliferation, survival and migration of cancer cells [67]. Moreover, lipids play a key role in macrophage polarization. During cancer treatment, the proliferation and migration of cancer cells can be greatly inhibited by blocking fatty acid uptake and oxidation to induce M2 to M1 conversion in TAMs [68]. Moreover, lipids are known to influence interactions between tumor cells and TAMs at every stage of cancer progression [69,70]. In the present study, we investigated the effect of the lipids PA, SM, Cer and DHA on breast cancer cells and TAM polarization (Table 1). Our data show that PA, SM, DHA and Cer not only decreased the migration and invasiveness of breast cancer cells, but also promoted the polarization of M2 macrophages towards the M1 phenotype with anti-tumor properties. Although the CM of M2-polarized macrophages promoted 4T1 cell proliferation, migration and invasion, treatment of M2 macrophages with PA, DHA and Cer reduced the pro-tumorigenic properties of the CM. In addition, exogenous uptake of PA and SM induced cellular toxicity in breast cancer cells. Overall, the anti-tumor properties of PA were most prominent among the screened lipids. Our results show that PA not only inhibited the migration and invasiveness of 4T1 breast cancer cells, but also reduced their proliferation and cell viability in a dose-dependent manner. Low (30 μM) and high doses of PA (100 μM) promoted apoptosis in 4T1 cells. Our findings are consistent with previous studies. For example, Zafaryab et al., showed that PA reduced the viability and induced apoptosis in the breast cancer cell line MCF-7 [45]. Similarly, previous studies by Baumann et al. have shown that PA activates the endoplasmic reticulum stress response network in breast cancer cells, induces cell cycle delay and inhibits tumor cell growth [71]. When 4T1 cells were co-cultured with CM of M2 macrophages treated with 30 μΜ PA, the proliferation of 4T1 cells was significantly reduced compared with the respective control.

Ceramides are tumor suppressors involved in immunomodulation [72] and can inhibit cancer cell proliferation by blocking the cell cycle [73], or by triggering the autophagic response of cancer cells by downregulating mTOR activity [74]. A study by Chang et al. showed that Cer was selectively cytotoxic to cancer cells, but non-toxic to normal cells. In contrast, our study showed that Cer did not reduce the viability of 4T1 cells (and M0, M1-type and M2-type macrophages), but inhibited the proliferation and migration of breast cancer cells significantly. In addition, our previous and current studies showed that Cer promoted the repolarization of tumorigenic M2 type macrophages towards the pro-inflammatory M1 type [44]. In line with our results, Moro et al. found that the invasiveness of breast cancer cells was negatively correlated with the level of Cer in tissue samples of breast cancer, breast cancer metastasis and normal breast [75]. In a mouse model of liver cancer, Li et al. showed that Cer induced apoptosis in liver tumors by inhibiting the growth of TAMs and increasing T-cell signaling, thereby slowing tumor growth in mice [57]. Thus, our results are in line with findings in the literature and support the notion that Cer possesses anti-tumor and immunomodulating properties.

The metabolites of SM are involved in a variety of cellular processes, such as cell proliferation, differentiation, senescence, apoptosis and immune regulation [76,77]. There are contradicting reports in the literature on the effect of SM on cancer cells. Both pro-tumor and anti-tumor properties have been reported in different types of cancer [39,42,78]. A study by Yan et al., demonstrated that in a triple-negative breast cancer mouse model, SM inhibited the proliferation and metastasis of cancer cells by reversing the polarization of M2 macrophages in tumors [79]. An earlier study by Modrak et al. found that SM promotes apoptosis in human pancreatic cancer cells [80]. A recent study identified SM as a key secreted bioactive factor from mammary cells that caused triple-negative breast cancer cell death in vitro and reduced tumorigenicity in a xenograft mouse model in vivo. In addition, SM induced significant downregulation of genes associated with breast cancer progression [38]. These findings are consistent with our experimental results, showing that SM and a CM of M2 macrophages treated with SM decreased breast cancer cell viability, proliferation and migration. In addition, and in line with previous results, SM significantly increased the M1 phenotype in lipid-treated M2-differentiated macrophages.

DHA is cytotoxic to many types of cancer cells. There is much evidence that DHA promotes cell cycle arrest and reduces the expression of cell cycle markers to inhibit cell proliferation in 4T1 mouse mammary cells and MCF-7 human breast cells [81,82]. It was shown that DHA could reduce the invasive potential of the MDA-MB-231 breast cancer cell line [83]. Rahman et al. showed that DHA effectively inhibited the growth and metastasis of breast cancer cells, possibly by blocking the migration of cancer cells to the bone. In addition, DHA was reported to enhance the inhibitory effect of doxorubicin on proliferation and invasion of MCF-7 cells [52]. *n*-3 poly-unsaturated fatty acids, such as DHA, have been shown to selectively inhibit tumor cell proliferation, without inducing cytotoxicity in normal cells [84]. While our results confirm the anti-tumor properties of DHA by reducing cell viability, proliferation and migration of breast cancer cells, DHA also reduced the viability of macrophages at concentrations above 75 μM. Furthermore, our experimental data show that low concentrations of DHA (to avoid cellular toxicity) did not significantly affect the macrophage polarization status, while other studies reported immunomodulatory properties of DHA [85,86,87]. Notably, Rajasinghe et al. demonstrated that the physiological concentration of DHA (25 μM) not only significantly enhanced macrophage phagocytosis, but also protected macrophages from crystalline silica-induced cell death [88]. These results suggest that the cellular effects of DHA might be partially dependent on the DHA concentration.

For most cancers, progression to malignancy is accompanied by EMT, which promotes cancer cell invasion and metastasis in a variety of cancers [89,90]. EMT is a dynamic process in cancer cells, and factors released by TAMs or immune cells in the TME can promote EMT and changes of expression in a variety of molecules [91,92], such as E-cadherin, N-cadherin and vimentin. EMT-associated transcription factors, such as Snail, zinc-finger E-box-binding (ZEB), Slug, Smad, and basic helix-loop-helix transcription factors, are thought to play a key role in regulating the expression of EMT markers [93,94]. For example, Snail has been shown to promote EMT by suppressing E-cadherin and promoting vimentin expression [95]. Cavalcante et al., demonstrated that inhibition of the STAT3/NF-κB signaling axis can effectively disrupt the interaction between macrophages and malignant cells, thereby reducing the immunosuppressive response [96]. Our results show that PA, SM, DHA and Cer could reduce EMT of breast cancer cells directly, as well as by partially inhibiting the CM-promoted increase in EMT. This was accompanied by the reduction of EMT marker vimentin, and an increase in E-cadherin expression. At the molecular levels, this coincided with the downregulation of *Nfkb1*, *Snai1* and *Stat3* mRNA, in line with our previous study showing that PA and Cer were powerful inhibitors of an IL-10-STAT3-NF-κB signaling axis regulating EMT in colorectal cancer [44]. Interestingly, our study demonstrates that all of the tested lipids partially overruled the pro-proliferative and pro-migratory effects of the M2 macrophage CM. Overall, our findings are in agreement with other studies that reported anti-tumor properties of Cer, PA, SM and DHA by either inhibiting the pro-tumor effects of M2 macrophages, or by acting on tumor cells.

## 4. Materials and Methods

### 4.1. Materials and Reagents

Fetal bovine serum (FBS), Iscove’s modified Dulbecco’s medium (IMDM), Dulbecco’s Modified Eagle’s Medium (DMEM) and Roswell Park Memorial Institute (RPMI) 1640 Medium were purchased from Gibco Laboratories (Thermo Scientific™, Waltham, MA, USA). For ELISAs, the following antibodies (Abs) were used: purified anti-mouse IL-10, anti-mouse IL-12/IL-23 p40 (monomer, dimer, heterodimer), biotinylated anti-mouse IL-10 and biotinylated anti-mouse IL-12/IL-23 p40 (monomer, dimer, heterodimer), all purchased from BioLegend (San Diego, CA, USA). Cell Titer 96^®^ Aqueous MTS Reagent Powder was purchased from Promega (Madison, WI, USA). DAPI (4′,6-diamidino-2-phenylindole, dilactate), goat anti-rabbit IgG (H+L) secondary Ab, Alexa Fluor 488, goat anti-rat IgG (H+L) secondary Ab Alexa Fluor 647, Hoechst 33342, CD324 (E-Cadherin) monoclonal Ab (clone DECMA-1) and anti-vimentin Ab (clone V9) were purchased from Thermo Fisher Scientific (Waltham, MA, USA). Anti-mouse CD163-PerCP-eFluor™ 710, anti-mouse CD68-FITC, anti-mouse CD86-FITC and anti-mouse/rat Ki-67 APC were obtained from eBioscience (San Diego, CA, USA). SM (Brain, Porcine) and Cer (porcine, brain) were purchased from Avanti Polar Lipids (Alabaster, AL, USA) and PA was purchased from Sigma-Aldrich (Darmstadt, Germany). DHA was purchased from Sigma-Aldrich (Darmstadt, Germany). Recombinant mouse IL-4, recombinant mouse IL-13, IFN-γ and lipopolysaccharide (LPS) were purchased from PeproTech (Rocky Hill, NJ, USA).

### 4.2. Cells

Samples of 4T1 (ATCC^®^ CRL2539™) murine breast cancer cells and mouse macrophages RAW 264.7 (ATCC^®^ TIB-71™) (mouse mononuclear macrophage cells) used in this study were purchased from the American Type Culture Collection (ATCC) (Manassas, VA, USA).

Samples of 4T1 murine breast cancer cells were cultured in complete RPMI1640 medium containing 10% FBS, 1% penicillin (P) and 1% streptomycin (S). The murine macrophage cell line RAW 264.7 was cultured in complete DMEM medium supplemented with 1% P/S and 10% FBS. Both cell lines were cultured in an incubator with 5% CO_2_ at 37 °C.

### 4.3. Preparation, Culture of Bone Marrow-Derived Macrophages (BMDMs)

Bone marrow cells were isolated from the femur and tibia of 6–8-week-old C57BL/6 mice [97,98]. Briefly, after disinfecting the skin of the mice with 70% alcohol, the muscle attached to the bone was removed using sterile scissors and forceps and the femur and tibia were placed in ice-cold PBS. The bone marrow was then flushed into ice-cold PBS using a 21G needle and 5 mL syringe. Afterwards, solid debris was removed by passing the cells through a 70 μm filter. The filtrate was centrifuged at 400× *g* for 10 min at 4 °C. The supernatant was removed and erythrocytes were lysed by resuspension in erythrocyte lysis buffer. Subsequently, the cells were incubated in complete DMEM at 37 °C for 4 h. The supernatant was collected and centrifuged at 400× *g* for 10 min at 4 °C, resuspended in complete DMEM containing 15% L929, and incubated at 37 °C with 5% CO_2_ for 7 days. The cell population was analyzed by flow cytometry for F4/80 and CD11b expression [97].

### 4.4. Preparation of Lipids

The lipids were dissolved in ethanol to prepare an ethanolic lipid solution. Briefly, ethanol was added to the lipid powder and dissolved at room temperature (RT) for 5 min followed by sonication for 3 min to achieve complete dissolution. Then, a lipid stock was prepared by mixing the ethanol lipid solution with fatty acid-free BSA. The stock was stored in glass vessels in a −20 °C. At the same time, an equal volume of ethanol was used in the preparation of ethanol: BSA mixture, which served as the control group for the experiments.

### 4.5. Polarization of RAW 264.7 Cells towards M1 and M2-Macrophages

For differentiation of RAW 264.7 cells into M1-polarized macrophages, RAW 264.7 cells were cultured in complete medium (10% FBS, 1% P/S) with 1 μg/mL LPS and 40 ng/mL IFN-γ for 24 h at a concentration of 2 × 10^5^ cells/well plated in a 24-well plate. For differentiation of RAW 264.7 cells into M2-polarized macrophages, 20 ng/mL IL-4 was used in place of LPS and IFN-γ. After treatment, cells were washed three times with PBS and cultured in serum-free (SF) DMEM medium supplemented with IL-4, or IFN-γ and LPS for another 48 h to obtain M1- and M2-polarized TAMs. The supernatant of M2-polarized TAMs was collected and stored in a refrigerator at −20 °C, and later used in our experiments as a conditioned medium (CM).

For differentiation of BMDMs into M2-polarized macrophages, BMDMs were cultured in complete medium (10% FBS, 1% P/S) with IL-4 (20 ng/mL) and IL-13 (20 ng/mL) [97].

In order to explore the effect of lipids on the polarization state of M2 macrophages and M1 macrophages, the cells were cultured as described above. After 24 h, the cells were washed three times with SF medium and resuspended in SF medium substituted with different concentrations of lipids (10, 25, 50, 75, 100, 150 and 200 μM, or, 10, 30 and 100 μM). After 48 h in the presence of lipids, the cells were collected, and the supernatants were stored and later used as a CM of lipid-treated macrophages (CM+).

### 4.6. Cell Viability Assays

The effects of PA, Cer, DHA, and SM on the metabolic activity of 4T1 cells, RAW 264.7 cells, M1 cells, and M2 cells were measured by an MTS assay, according to the manufacturer’s instructions. Cells were cultured and treated with lipids as described above. Then, the culture medium was discarded and 100 μL of fresh culture medium and 20 μL/well of MTS reagent were added to each well and incubated for 2 h a 37 °C in an incubator. Absorbance was measured at 490 nm with a microplate reader (BioTek, Winooski, VT, USA). 40% DMSO was used as a death control group, the serum-containing medium was used as a positive control group, and SF and drug-free media were used as a viability control. Wells without cells were used as the blank control group.

### 4.7. Flow Cytometry

Flow cytometry was used to detect the effect of lipids on the characteristics of M1 or M2 macrophages and the proliferation of 4T1 cells. Briefly, after incubation of M1 and M2 macrophages with different concentrations of lipids for 48 h, cells were washed twice, detached from 24-well plates by trypsinization and resuspended in a FACS buffer containing Abs for extracellular labeling. To assess the effect of lipids on the expression of M1 or M2 macrophage markers, cells were labeled with anti-CD86 (1 μg/mL), anti-CD68 (1 μg/mL) and anti-CD163 (1 μg/mL) Abs. To assess the effect of lipids and CM+ on 4T1 cell proliferation and migration, cells were labeled with anti-E-cadherin (1 μg/mL) and anti-vimentin (1 μg/mL) Abs.

For intracellular Ab labeling, cells were first fixed in 4% paraformaldehyde (PFA) for 10 min at RT, followed by permeabilization in intracellular staining permeabilization buffer (Biolegend) for 10 min, then incubated with 100 uL intracellular staining buffer containing anti-mouse CD68-FITC Ab (1 μg/mL) or Ki-67 (1 μg/mL) on ice for 45 min. After washing with PBS, samples were resuspended in 100 μL PBA and measured by flow cytometry (BD LSR III, Bioscience). For each sample, at least 10,000 events were acquired and analyzed using FlowJo 10.1 software.

### 4.8. Immunofluorescence

Samples of 4T1 cells were seeded on coverslips with a diameter of 13 mm at a density of 3 × 10^4^ per well in a 24-well plate, and cultured overnight. According to the experimental requirements, 4T1 cells were cultured in an SF medium containing 30 μM PA, Cer, SM and 10 μM DHA. After 48 h, 4T1 cells were fixed for 15 min with 1% PFA. Then, after washing with PBS, cells were permeabilized with 0.1% Triton X-100 in PBS for 10 min, followed by a washing step and blocking in a blocking solution (0.05% Tween-20 and 5% normal goat serum in PBS) for 30 min at RT. The cells were labeled with rabbit Ab against E-cadherin (2 μg/mL) and rat Ab against vimentin (2 μg/mL) in 0.1% BSA/PBS and incubated at 4 °C overnight. After 24 h, the samples were washed 3 times with washing solution, and then labeled with goat rabbit IgG Alexa Fluor^®^ 488 and goat rat IgG Alexa Fluor^®^ 647 for 1 h at RT according to the manufacturer’s description. Finally, the cells were washed and the cell nuclei were labeled with DAPI for 5 min at RT. After washing in PBS, the samples were mounted in a fluorCare mounting medium. The samples were imaged using an immunofluorescence microscope (Leica DM5500 B, Leica Microsystem CMS GmbH, Mannheim, Germany) and analyzed by LAS X (Leica Application Suite X, CMS GmbH, Mannheim, Germany) software.

### 4.9. In Vitro Migration Assays

Tumor cells were seeded at 2 × 10^4^ cells per well in IncuCyte^®^ ImageLock 96-well plates (Sartorius Group, Essen BioScience, Ltd., Newark CI SG8 5HL, UK) and incubated for 24 h. When the cell density reached 95%, the IncuCyte^®^ Cell Migration Kit (BioScience, Ltd., Newark CI SG8 5HL, UK) was used to draw a “wound”. The cells were subsequently treated with 10 μg/mL mitomycin in SF medium for 1.5 h at 37 °C to inhibit cell proliferation. Then, the cells were washed twice with the SF medium, and subsequently cultured in medium supplemented with 30 μM PA, Cer, SM or 10 μM DHA, or the CM of M2 macrophages treated with 30 μM PA, Cer, SM, or 10 μM DHA. The wound healing process was monitored every 2 h for 48 h, and the wound healing rate was calculated using the IncuCyte^®^ Live-Cell Analysis System.

### 4.10. Transwell Migration Assays

The 4T1 cell migration assay was performed using a Transwell cell culture chamber (Corning Costar, Corning, NY, USA). Briefly, 4T1 cells were incubated with 30 μM PA, Cer, SM or 10 μM DHA, or the CM of M2 macrophages treated with 30 μM PA, Cer, SM, or 10 μM DHA, respectively. After 48 h, 1 × 10^4^ 4T1 cells were inoculated into the upper chamber of a 24-well transwell plate (pore size 8 μm) and incubated in full DMEM medium for 16–18 h. Then, the inset was removed and the cells at the bottom of the transwell plate were collected, resuspended in trypan blue and counted using a TC20 Automated Cell Counter (Biorad, Hercules, CA, USA).

### 4.11. Enzyme-Linked Immunosorbent Assay (ELISA)

M2-differentiated RAW 264.7 macrophages were incubated for 48 h in the presence of different concentrations of lipids. Subsequently, the cells were centrifuged and the cell culture supernatant was collected and stored in a refrigerator at −20 °C until further analysis. ELISA was performed according to the manufacturer’s recommendations. Absorbance was measured using a microplate reader (Tecan, Reading, UK,) at 450 nm. All samples were run in duplicate and analyzed using the average values.

### 4.12. Quantitative Real-Time PCR

According to the manufacturer’s instructions, total RNA was extracted from cell samples using Trizol (Invitrogen; Carlsbad, CA, USA). Then, the RNA concentration was measured by Nanodrop spectrophotometry. cDNA was synthesized using the M-MLV Reverse Transcriptase (Promega, Madison, WI, USA). With Gapdh as the endogenous control group, the expression levels of *Stat3*, *Snai1* and *Nfkb1* were analyzed by the Step One Plus real-time PCR detection system (Bio-Rad, Hercules, CA, USA). The data of each gene was normalized to the Gapdh gene, and the fold change was calculated by relative quantification (2^−ΔΔCT^). The following primers were used: *Gapdh*: forward primer, 5′TGGTGAAGCAGGCATCTGAG3′ and reverse primer, 5′TGAAGTCGCAGGAGACAACC3′. *Stat3:* forward primer, 5′CTTGTCTACCTCTACCCCGACAT3′ and reverse primer, 5′GATCCATGTCAAACGTGAGCG3′. *Snai1*: forward primer, 5′TCTGAAGATGCACATCCGAAGCCA3′ and reverse primer, 5′AGGAGAATGGCTTCTCACCAGTGT3′. *Nfkb1:* forward primer, 5′GAAATTCCTGATCCAGACAAAAAC3′ and reverse primer, 5′ATCACTTCAATGGCCTCTGTGTAG3′.

### 4.13. Statistical Analyses

Statistical analyses were performed using GraphPad Prism software version 8.0.1 by an unpaired *t*-test followed by the Mann-Whitney test or two-way ANOVA followed by a Bonferroni test to calculate the significance of the difference between two groups. The experimental data are expressed as mean ± SEM.

## 5. Conclusions

In summary, our study demonstrates that the different types of natural lipids, PA, SM, Cer and DHA possess several anti-tumor properties, namely the simultaneous inhibition of the cellular proliferation, migration and invasiveness of cancer cells. In addition, SM, Cer, PA and DHA promoted (to different extents) the conversion of M2 macrophages to the pro-inflammatory type M1. Overall, the anti-tumor actions of PA were most prominent, as in addition to diverse anti-tumor properties, PA also induced apoptosis of cancer cells. This opens up new opportunities for PA as an anticancer agent for the treatment of breast cancer. The literature also provides evidence that PA shows effectiveness in anti-tumor treatment in vivo. For example, Lin et al. showed that food supplementation with PA inhibited the growth of liver tumors in tumor-bearing nude mice and reduced the rate of hepatocellular carcinoma lung metastasis [99]. The current work highlights a potentially powerful role of PA in reducing the tumorigenic properties of breast cancer cells, while simultaneously reducing the immunosuppressive mechanism of TAMs. Thus, PA may provide a new strategy for dual macrophage- and tumor-targeted anti-cancer therapies.

## Figures and Tables

**Table 1:** Summary of the anti-tumor and immunomodulatory effects of PA, SM, Cer and DHA on 4T1 cells and M2-polarized macrophages, respectively.

Effect on:	4T1								M2 TAMs				
	Viability	Apoptosis	Proliferation	E-cadherin	Vimentin	*NF* *κB*	*S* *nai1*	*S* *tat3*	CD163	CD86	CD68	IL10	IL12
PA/CM+PA	+	+	+	+/+	+/+	+/+	+/+	+	+	+	+		+
SM/CM+SM	+		+/+	+	+/+	+	+			+	+	+	+
Cer/CM+Cer			+/+	+	+	+	+/+		+/+	+		+	+
DHA/CM+DHA			+/+		+/+	+/+	+/+		+/+	+			+

## Data Availability

The original contributions presented in the study are included in the article/supplementary material. Further inquiries can be directed to the corresponding author.

## Supplementary Materials

The following supporting information can be downloaded at: https://www.mdpi.com/article/10.3390/ijms23084240/s1.
