# Supplementary material for: Multifunctional Role of Lipids in Modulating the Tumorigenic Properties of 4T1 Breast Cancer Cells
Source: Int J Mol Sci. 2022 Apr 11;23(8):4240. doi: 10.3390/ijms23084240 (PMC9024985; doi:10.3390/ijms23084240)
Supplement: Supplementary file 1 [file ijms-23-04240-s001.zip › ijms-1635269-supplementary.pdf]

International Journal of
Molecular Sciences

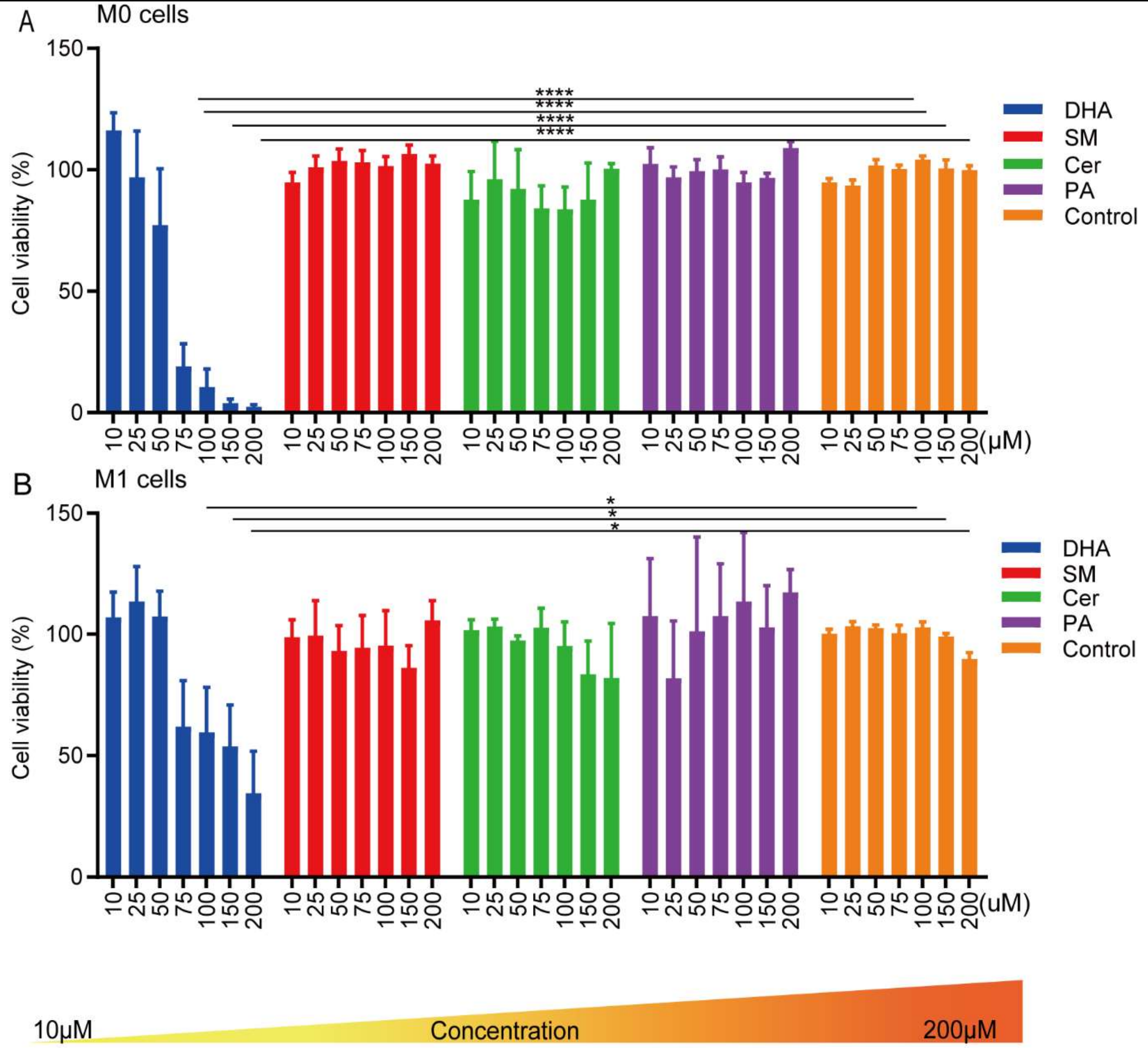

Figure S1. Cell viability of (**A**) M0 and (**B**) M1 cells after treatment with SM, DHA, Cer or PA for 48 h. M0 and M1-macrophages were treated with increasing concentrations (10–200 μM) of SM, DHA, Cer, or PA. After 48 h, the cell viability was assessed by MTS assay. The data represent the mean ± SEM of 6 independent experiments. All p-values were compared to the respective control cells using Two-way ANOVA test. * $p \leq 0.0272$; **** $p \leq 0.0001$.

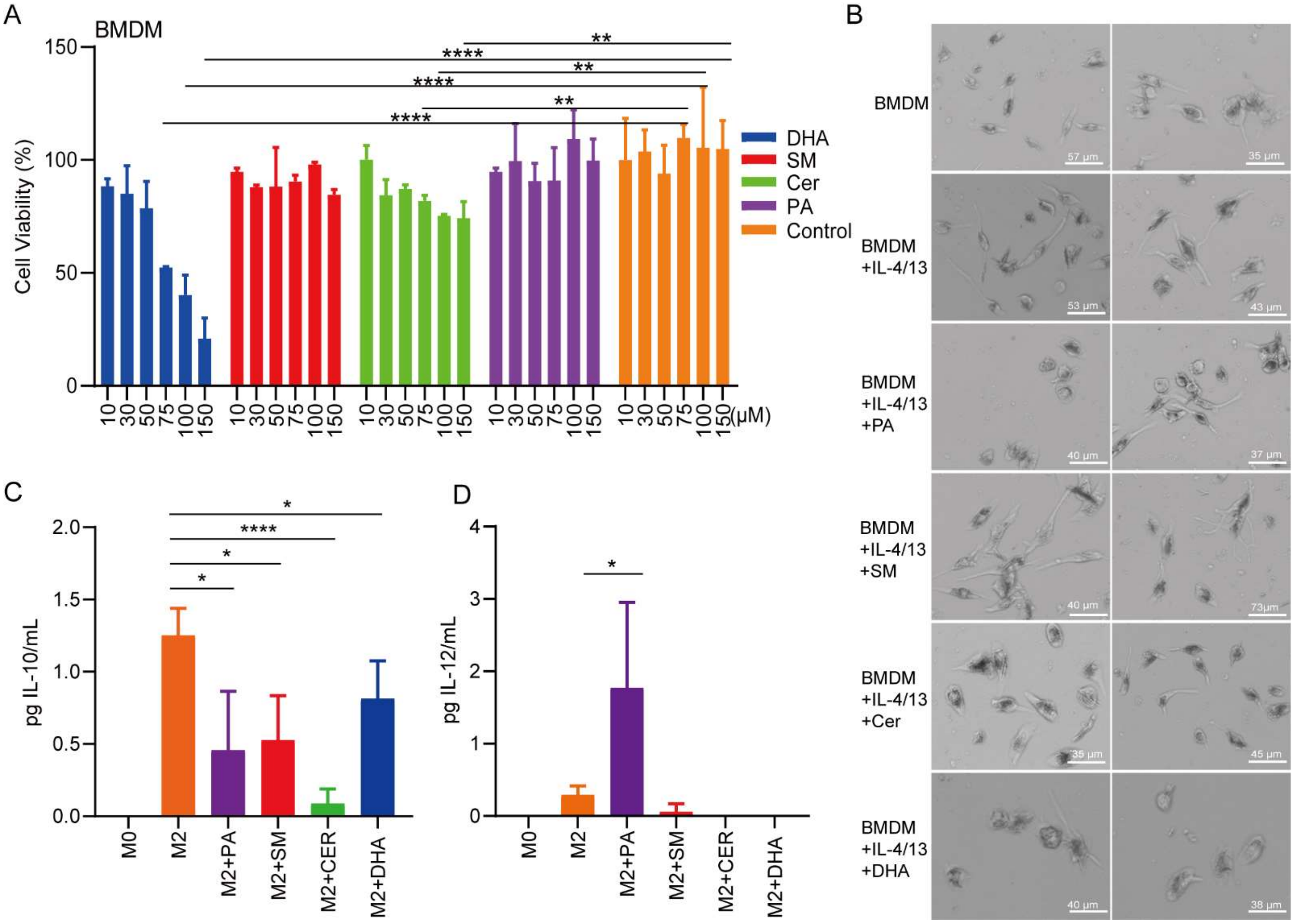

Figure S2. Response of bone marrow derived macrophages (BMDMs) towards lipids. (**A**) Cell viability of BMDMs after treatment with increasing concentrations (10–150 μM) of SM, DHA, Cer, or PA. After 48 h, the cell viability was assessed by MTS assay. The data represent the mean ± SD of one representative experiment. All p values were compared to control cells by two-way ANOVA and the Bonferroni's post hoc test. (**B**) Representative morphological appearance of control and IL-4/13 polarized BMDMs, in the absence or presence of 10 μM DHA, 30 μM PA, 30 μM SM, or 30 μM Cer. The M2 and M1 cytokines IL-10 (**C**) and IL-12 (**D**), respectively, were quantified in supernatants of IL-4/13-polarized BMDM cells treated with lipids by ELISA. The data represent the mean ± SD. All p values were compared to control cells by unpaired t test. * $p \leq 0.05$; ** $p \leq 0.01$, **** $p \leq 0.0001$.

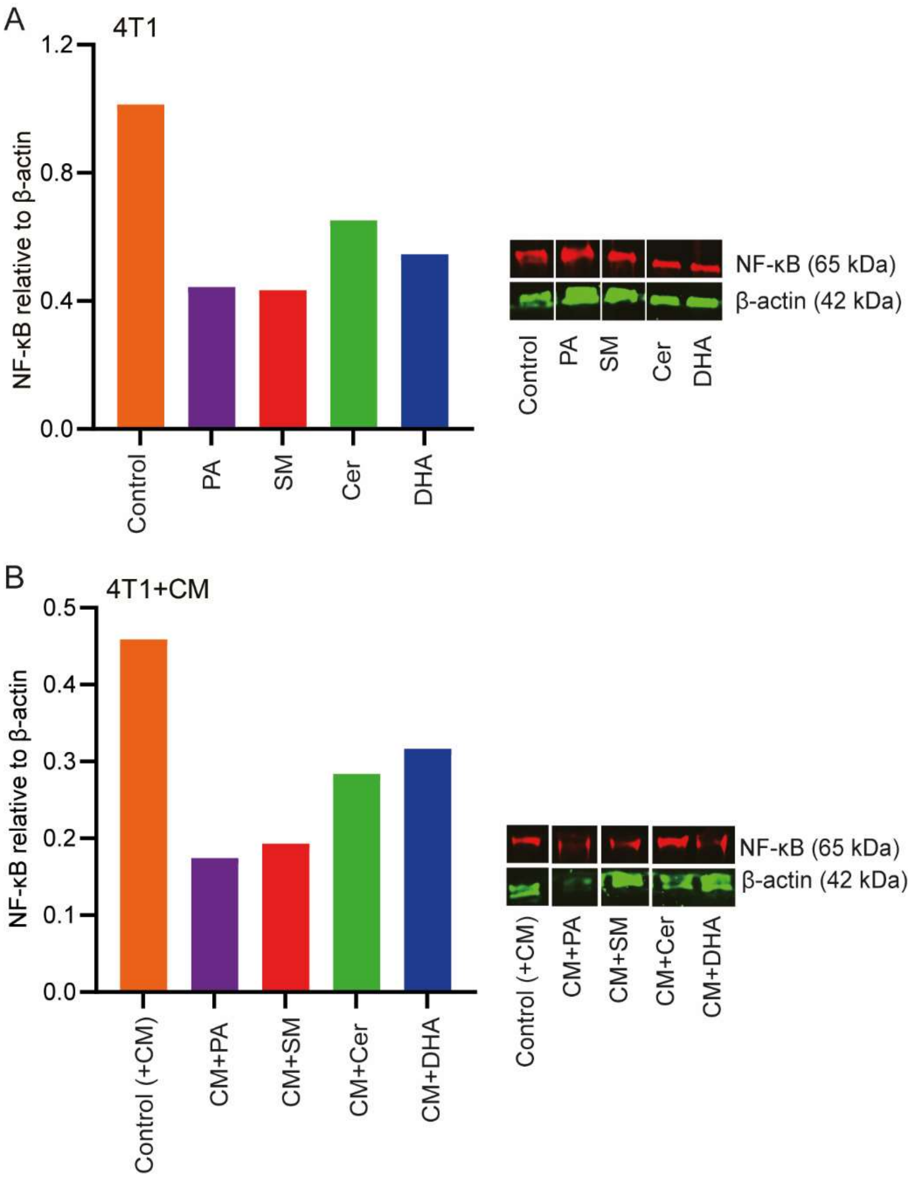

Figure S3. Expression of NF-κB (red) and β-actin (green) protein in 4T1 cells treated with (**A**) 30 μM PA, 30 μM SM, 30 μM Cer or 10 μM DHA, or (**B**) CM+ 30 μM PA, 30 μM SM, 30 μM Cer or 10 μM DHA by western blot labeling. The signal intensities of the NF-κB and β-actin bands were quantified using ImageJ software. The NF-κB signal is displayed relative to the signal obtained for β-actin.
